# Diagnostic challenges and surgical options for cysts of the canal of Nuck in adults: Two cases report

**DOI:** 10.1097/MD.0000000000041980

**Published:** 2025-04-25

**Authors:** Gai Hang, Huakang Wang, Yuyang Wang, Quan Wen, Zhiyu Yu, Yunpeng Guo, Bo Chen

**Affiliations:** a Tongliao People’s Hospital of Inner Mongolia Autonomous Region, Tongliao, China; b Huangpi District People’s Hospital of Wuhan, Huangpi District, Wuhan City, Hubei Province, China; c Inner Mongolia Medical University, Huhehot, China.

**Keywords:** canal of Nuck, hydrocele, laparoscopic surgery

## Abstract

**Rationale:**

Cyst of the canal of Nuck is a very rare female hydrocele, which is often caused by developmental disorders. The incidence of this disease is very low in the world, and misdiagnosis and missed diagnosis often occur due to insufficient understanding of it by clinical staff. We present these 2 case studies with the objective of enhancing awareness and comprehension of the disease, as well as offering valuable insights for its management and treatment.

**Patient concerns:**

A 38-year-old female patient was admitted to the hospital with a reversible mass in the left groin area that was painful during the menstrual period.A 50-year-old emergency patient who was admitted to the hospital for the discovery of an incarcerated mass in the left groin area for 2 hours.

**Diagnoses:**

In case 1, inguinal hernia was considered before operation, and the canal Nuck type I complicated with hernia was found intraoperatively.In case 2, cyst of the canal of Nuck was considered before operation, and the canal Nuck type Ⅲ was found intraoperatively.

**Interventions:**

In case 1, the patient was given tension-free hernioplasty combined with resection of a cyst of the round ligament of uterus. In case 2, the patient underwent transabdominal preperitoneal prosthesis plus cyst dissection of the round ligament of uterus.

**Outcomes:**

In both patients, the mass in the groin area was completely removed and the pain caused by the mass disappeared.

**Lessons:**

These 2 cases emphasize that the possibility of hydrocele of the canal Nuck should be taken into account when discovering a female groin mass. Ultrasound, computed tomography, and magnetic resonance imaging are conducive to diagnosis, but the diagnosis also needs intraoperative findings and postoperative pathology. For this disease, surgical treatment is the first choice.For cyst of the canal of Nuck type I, transabdominal preperitoneal patch implantation is recommended. When cyst of the canal of Nuck type III is found under laparoscopy and it is difficult to simply use laparoscopic dissection, a small auxiliary incision can be made in the inguinal region to preserve the round ligament of the uterus and completely peel off the cyst.

## 1. Introduction

Hydrocele of the canal Nuck is a very rare developmental disorder. According to world health organization, the prevalence of this disease in girls aged 0 to 16 years is only 0.76 %.^[1]^ There are fewer reports on cyst of the canal of Nuck in adult women, only in case reports. Coley first reported hydrocele of the canal Nuck in women in 1892.^[2]^ Up to now, only about 50 publications have been searched using PubMed for the topic of hydrocele of the canal Nuck in adult women.The clinical manifestations of this disease are similar to those of inguinal hernia. It is mainly manifested as a mass from the inguinal region to the labia. Its essence is a pocket-shaped protrusion formed when the intraperitoneal fluid enters the parietal peritoneum and descends to the female labia along the inguinal canal along the inguinal ring through the round ligament of the uterus. Therefore, it can also be called ``female hydrocele.’’ When there are obvious symptoms, obvious enlargement of the tumor, rapid enlargement or suspicious factors in the auxiliary examination, active surgical treatment should be carried out. Otherwise, due to the traction of the round ligament of the uterus, it can cause the ovary and fallopian tube to protrude into the abdominal cavity, complicated with indirect inguinal hernia, and easily cause the torsion and necrosis of the ovarian mesentery. Due to the lack of basic understanding of its diagnosis and treatment by clinical first-line doctors, misdiagnosis and incomplete treatment are more likely to occur. Therefore, we retrospectively analyzed 2 cases of hydrocele of the canal Nuck admitted to our hospital, as a case study here.

## 2. Case report

**Figure 1.**
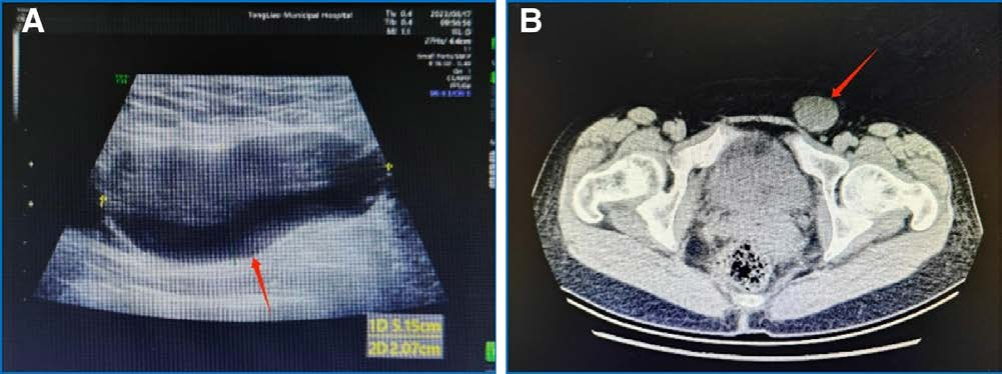
(A) Ultrasonography showed a cystic mass, smooth cyst wall, low echo signal, and no detectable blood flow signal (red arrow). (B) CT showed a clear, regular, low-density cystic mass in the left groin area (red arrow). CT = computed tomography.

**Figure 2.**
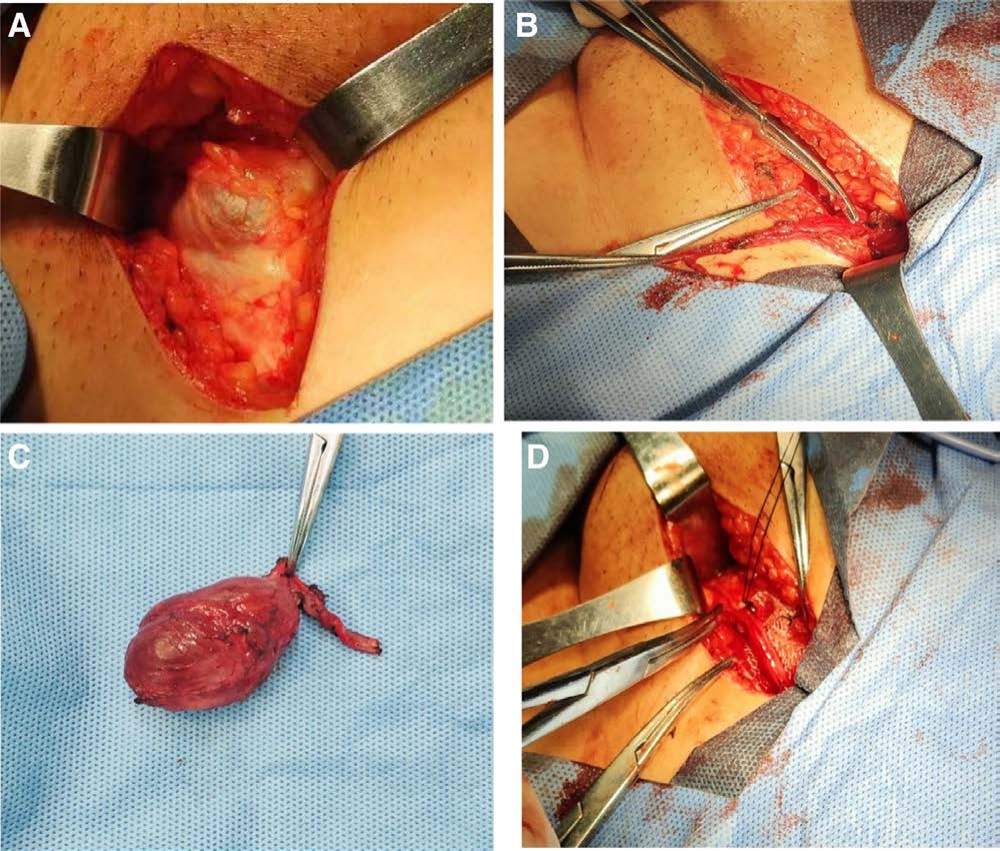
(A) Exfoliate the omentum attached to the wall of the hernia sac. (B) Ligate the neck of the hernia sac at the high level of the internal annulus. (C) The cyst was removed completely after freeing the round ligament of uterus. (D) A patch was placed behind the round ligament of the uterus to strengthen the posterior wall of the groin.

*Case 1*: A 38-year-old patient with a history of intestinal obstruction surgery was admitted to the hospital with a left groin reversible mass. She reported that the lump were painful and related to the menstrual cycle. During physical examination, the patient stood and saw a left groin mass with soft quality and slight tenderness. The left inner ring mouth was about one finger tip wide. When patient coughing, there was a sense of impact. The light transmittance test was negative, and it could disappear in the supine position. Color Doppler ultrasound examination in our hospital showed a cystic mass of 5.15 × 2.07 cm in the left groin area. The cystic fluid was clear, the cystic wall was smooth, and no blood flow signal was detected (Fig. 1A). Consider inguinal hernia. Because of its previous history of intussusception surgery and the hernia sac away from the outer ring, we adopted tension-free hernioplasty. During the operation, it was found that Nuck cyst typeⅠwas combined with hernia, so the resection of uterine round ligament cyst was performed at the same time. We stripped the greater omentum that adhered to the wall of the hernia sac (Fig. 2A), ligated the hernia sac neck at the high position of the inner ring(Fig. 2B), and completely removed the cyst after freeing the round ligament of the uterus (Fig. 2C). A patch was placed behind the round ligament of the uterus to strengthen the posterior wall of the groin (Fig. 2D) and sutured layer by layer. Histopathological examination showed a cyst of the left round ligament of the uterus, and there was no obvious abnormality after 3 months of follow-up.

**Figure 3.**
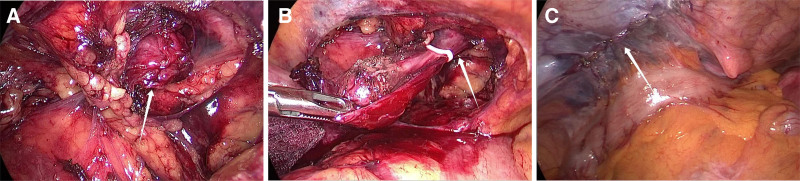
(A) Hydrocele of the canal Nuck type Ⅲ was incarcerated at the internal annulus. (B) The neck of the cyst was clamped to remove the cyst completely. (C) The peritoneum was sutured with a patch placed below the internal annulus.

**Figure 4.**
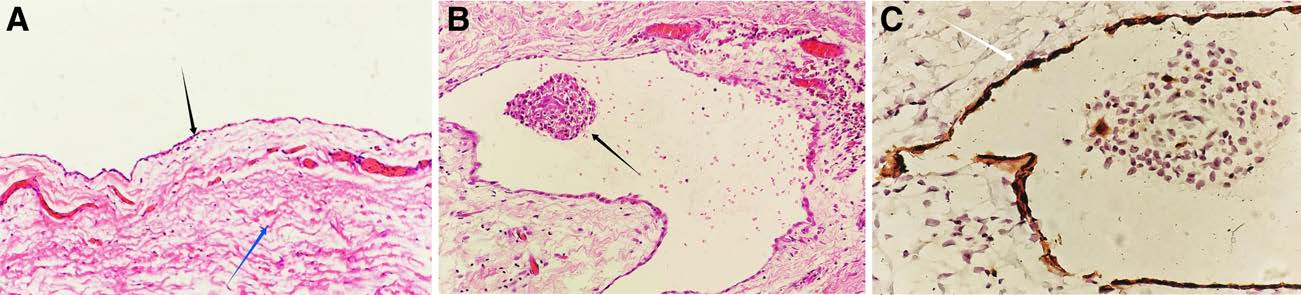
(A) Flat mesothelial cells are seen in bundles on the wall of the capsule (black arrow), and some tissues contain smooth muscle fiber structures (blue arrow). (B) Lymphocyte infiltration is seen around the cyst wall (black arrow). (C) Immunohistochemical staining of mesothelial cells and columnar epithelial cells was positive for calretinin (white arrow).

*Case 2*: A 50-year-old emergency patient who was admitted to the hospital for the discovery of an incarcerated mass in the left groin area for 2 hours. The mass was about 5cm in size and had no tenderness. The patient had slight abdominal distension, no abdominal pain, and the mass could not disappear in the supine position, nor could it be restored manually. Color Doppler ultrasound examination revealed a tubular cystic anechoic mass of about 10.24 cm × 2.66 cm in size in the left groin area, with regular shape, a small separation in the inner wall, and no obvious blood flow signal. Computed tomography examination revealed a cystic mass of 7.1 cm × 2.9 cm × 2.6 cm in the left groin area, with clear boundary, regular shape, and communication with the abdominal cavity (Fig. 1B). Hydrocele of the canal Nuck was considered before operation, and selective laparoscopic transabdominal preperitoneal prosthesis and uterine round ligament cyst dissection was performed. Hydrocele of the canal Nuck type III was found during the operation (Fig. 3A). The cyst was separated from the round ligament of uterus, and the cyst neck was closed with a vascular clamp, thus the cyst was completely resected (Fig. 3B), and finally the patch was inserted 6 cm below the opening of the internal ring (Fig. 3C). Postoperative histopathology confirmed the left uterine round ligament cyst. After hematoxylin-eosin staining, flat mesothelial cells were arranged in bundles in the cyst wall, and some tissues contained smooth muscle fiber structure (Fig. 4A). Lymphocyte infiltration was observed around the cyst wall (Fig. 4B). Immunohistochemical staining of calretinin was positive in mesothelial cells and columnar epithelial cells (Fig. 4C). In both patients, the mass in the groin area was completely removed and the pain caused by the mass disappeared.

## 3. Discussion

The related nomenclature of hydrocele of the canal Nuck was first discovered by Dutch anatomist Anton Nuck in 1691.^[3]^ The Nuck tube is a kind of peritoneal sheath formed by the tubular folds of the round ligament of the uterus and the peritoneal wall extending into the groin. It is similar to the male vaginal process. When the peritoneal sheath is not closed or incompletely closed, the abdominal contents can enter the groin and the labia majora through the sheath. If the liquid passes through the narrow sheath, it forms a hydrocele of the canal Nuck. There are primary and idiopathic hydrocele of the canal Nuck. Primary refers to the defect of the secretory membrane of the processus vaginalis, which leads to an imbalance between the secretion and absorption of liquid.^[4]^ This imbalance may be caused by changes in lymph drainage caused by hormone levels, trauma or infection. However, most of the current cases are idiopathic hydrocele of the canal Nuck due to incomplete closure of the Nuck tube.^[5]^ This disease can be divided into 3 types. TypeⅠis the most common cyst of the canal of Nuck (also known as noncommunicating or cystic hydrocele) in clinical practice. It is unilocular cystic because it is not connected to the peritoneal cavity after cephalic closure. For type II, the size and shape of the cyst can change during the Valsalva test due to the continuous communication between the hydrocele and the peritoneal cavity.^[6]^ Type III is the most rare type, presenting as hourglass shape (also known as bilocular cyst of the canal of Nuck) due to narrow and deep inguinal ring compression of Nuck hydrocele.

Patients with cyst of the canal of Nuck often see a doctor for a removable or nonremovable mass in their groin area or upper labia minora. During physical examination, the abdomen of the patient may be palpable with a smooth surface, clear boundary, tough or cystic mass, or a pedicled mass located in the upper part of the labia minoris. The mass increases when standing or increasing abdominal pressure, and the signs are similar to inguinal hernia, and 40% of the patients with Nuck duct cyst also have inguinal hernia.^[7]^ In imaging, Nuck cyst was similar to inguinal lymphangioma, inguinal granuloma, endometriosis and other diseases, which undoubtedly made diagnosis more difficult. Just like case 1 here, the inguinal hernia was diagnosed by preoperative physical examination and color ultrasonography, but it was found that the hernia sac was actually adhered to around the cyst. In addition, some patients with this disease may experience swelling and pain associated with the menstrual cycle. Some scholars believe that this is related to the changes in the reproductive system during the menstrual cycle. Cysts increase in volume in a short period of time during menstrulation, resulting in the cyst’s volume continuing to increase after the deterioration of venous return. In addition, due to the concomitation of the genital branches of the ilioinguinal nerve and the genital femoral nerve, the enlarged cyst of the round ligament of the uterus causes tenderness.^[8]^ Some foreign scholars also believe that the presence of endometrial tissue in the vaginal process will lead to periodic swelling and discomfort related to menstruation. A similar phenomenon also appeared in case 1. The patient’s pain was related to the menstrual cycle. Combined with the postoperative pathology, the author believed that the patient’s pain and discomfort were mainly related to cyst infection and compression of the abdominal and femoral inner ring.

As imaging methods become more advanced, so does usability. As the most important preoperative auxiliary diagnosis method, imaging can observe the characteristics of the connection between the cystic mass with clear boundary and the round ligament of uterus.^[9]^ As the first choice for inguinal masses, high-resolution ultrasound can not only accurately distinguish inguinal masses from cystic or solid lesions, but also help timely diagnosis of intestinal obstruction and avascular necrosis.^[10]^ Some scholars have proposed that Valsalva movement in patients undergoing ultrasound examination can exclude or confirm the existence of inguinal hernia,^[11]^ but we have few patients, so the diagnosis rate has not been verified. Magnetic resonance imaging can show the relationship between cysts with low signal in T1-weighted imaging and high signal in T2-weighted imaging and surrounding tissues, which is more conducive to diagnosis, but it is not the first choice due to its high price.^[9]^

Long-term cyst of the canal of Nuck will relax the tissue structure around the inner ring and weaken the strength of the abdominal transverse fascia. Cyst of the canal of Nuck can cause the ovary and fallopian tube to protrude into the abdominal cavity through the pulling action of the round ligament of the uterus, so that the internal organs can enter the deep ring of the inguin, the lateral side of the inferior artery of the abdominal wall, and reach the labia major along the round ligament of the uterus. This kind of hernia is called “indirect” hernia. The most common hernia contents are the intestines and ovaries, which may lead to emergency conditions such as intestinal strangulation, intestinal obstruction and torsion and necrosis of the mesoovary. For patients with obvious abdominal pain or discomfort, we need to consider the above conditions before performing emergency surgery. Therefore, the correct diagnosis and timely treatment of uterine round ligament cyst are particularly important.

The treatment of cyst of the canal of Nuck is mainly surgical treatment, and the previously used cyst aspiration has a low cure rate and a high postoperative recurrence rate, so this operation is not recommended. At present, the commonly used surgical procedures include open lichtenstein, transabdominal preperitoneal patch implantation (TAPP), and total peritoneal patch implantation. Studies have shown that TAPP and lichtenstein are equivalent in the treatment of cyst of the canal of Nuck complicated with inguinal hernia, and the incidence of postoperative chronic groin pain is lower. Compared with total peritoneal patch implantation surgery, TAPP is easier to see anatomical markers and the operation time is shorter.^[12,13]^ Since the round ligament of uterus can maintain the forward position of uterus, and the genital branch of the genital femoral nerve is close to the round ligament of uterus, if the round ligament of uterus is cut off, it will lead to edema and numbness of the ipsolateral labia majora and some women’s poor sexual life experience, so we believe that attention should be paid to protecting the round ligament of uterus during the operation, especially for young female patients. Based on the literature review of cyst of the canal of Nuck cases in the past and the summary of surgical experience, it is recommended to choose TAPP for cyst of the canal of Nuck type I with less trauma. When the cyst of the canal of Nuck type III is seen in laparoscopy, and the cyst is too deep and the adhesion is serious, the whole laparoscopy can be abandoned, and another small auxiliary incision can be made in the inguinal region to preserve the round ligament of the uterus and completely peel the cyst.

To sum up, the possibility of cyst of the canal of Nuck should be taken into account when finding a female groin mass. Although ultrasound, computed tomography, and magnetic resonance imaging are conducive to diagnosis, the diagnosis also needs intraoperative findings and postoperative pathology. Surgical treatment is the first choice, but the best operation method should be selected for the patient according to the age, cyst type, and fertility requirements of the patient.

## Author contributions

**Investigation:** Yuyang Wang, Quan Wen.

**Supervision:** Zhiyu Yu, Yunpeng Guo.

**Writing – original draft:** Gai Hang.

**Writing – review & editing:** Huakang Wang, Bo Chen.
